# Alleles of *Insm1* determine whether RIP1-Tag2 mice produce insulinomas or nonfunctioning pancreatic neuroendocrine tumors

**DOI:** 10.1038/s41389-019-0127-1

**Published:** 2019-02-22

**Authors:** Shinta Kobayashi, Tanupriya Contractor, Evan Vosburgh, Yi-Chieh Nancy Du, Laura H. Tang, Richard Clausen, Chris R. Harris

**Affiliations:** 1 Institute for Advanced Study, Princeton, NJ USA; 2 Raymond and Beverly Sackler Foundation Laboratory, New Brunswick, NJ USA; 3 Weill Cornell Medicine, New York, NY USA; 4 Memorial Sloan Kettering Cancer Center, New York, NY USA; 5 Rutgers University Cancer Institute of New Jersey, New Brunswick, NJ USA; 6 Department of Pediatrics, Robert Wood Johnson Medical School, New Brunswick, NJ USA

## Abstract

The two most common types of pancreatic neuroendocrine tumors (PanNETs) are insulinomas and nonfunctioning PanNETs (NF-PanNETs). Insulinomas are small, rarely metastatic tumors that secrete high amounts of insulin, and nonfunctioning PanNETs are larger tumors that are frequently metastatic but that do not secrete hormones. Insulinomas are modeled by the highly studied RIP1-Tag2 (RT2) transgenic mice when bred into a C57Bl/6 (B6) genetic background (also known as RT2 B6 mice). But there has been a need for an animal model of nonfunctioning PanNETs, which in the clinic are a more common and severe disease. Here we show that when bred into a hybrid AB6F1 genetic background, RT2 mice make nonfunctioning PanNETs. Compared to insulinomas produced by RT2 B6 mice, the tumors produced by RT2 AB6F1 mice were larger and more metastatic, and the animals did not suffer from hypoglycemia or hyperinsulinemia. Genetic crosses revealed that a locus in mouse chromosome 2qG1 was linked to liver metastasis and to lack of insulin production. This locus was tightly linked to the gene encoding *Insm1*, a beta cell transcription factor that was highly expressed in human insulinomas but unexpressed in other types of PanNETs due to promoter hypermethylation. *Insm1*-deficient human cell lines expressed stem cell markers, were more invasive in vitro, and metastasized at higher rates in vivo when compared to isogenic *Insm1*-expressing cell lines. These data demonstrate that expression of *Insm1* can determine whether a PanNET is a localized insulinoma or a metastatic nonfunctioning tumor.

## Introduction

Neuroendocrine cells function by secreting hormones in response to neurological or metabolic stimuli. The insulin-producing beta cells of the pancreas are the best-known example of neuroendocrine cells, because beta cell defects can result in diabetes. Neuroendocrine cells are also found in many other sites of the body, including the pituitary, thyroid, parathyroid, small and large intestine. In order to maintain proper hormone balance, neuroendocrine cells are under tight growth regulation. However, neuroendocrine cells can become transformed and develop into neuroendocrine tumors. Transformation of pancreatic beta cells results in pancreatic neuroendocrine tumors (PanNETs). PanNETs are the second most common tumors of the pancreas, with an incidence of 1 per 200,000, and the incidence of PanNETs has been increasing rapidly^1^. PanNETs often metastasize to the liver.

For such an uncommon disease, PanNETs have been a surprisingly popular research subject for tumor biologists. This is partly due to the fact that PanNETs are produced by the RIP1-Tag2 tumor model (RT2), which was one of the very first transgenic mouse models for cancer^2^. PanNETs occur in RT2 mice due to expression of the SV40 T-antigen oncoprotein (Tag) from a rat insulin promoter (RIP). Tumor formation in RT2 mice is rapid and synchronized, which facilitates the testing of both potential therapeutics and potential tumor genes. RT2 is also a rare example of a mouse model that has been validated pharmacologically. Sunitinib and rapamycin were shown to block growth of tumors in RT2 mice^3,4^; these drugs were subsequently tested in clinical trials^5,6^, and approved by the FDA for use in patients. Conversely, antibodies against IGF1 receptor failed to block tumor progression in RT2 mice, and subsequently failed in the clinic^7,8^. The clinical success of RT2 as a model organism also prompted a reexamination of the Rb pathway in human PanNETs, because Rb is inactivated by the SV40 T-antigen. This analysis led to discovery of Cdk4 and Cdk6 amplifications and high Rb phosphorylation in pancreatic neuroendocrine tumors, as well as the demonstration that PanNET cell lines responded to Cdk4/6 inhibition especially in combination with rapamycin^9^. This study helped lead to a clinical trial of a Cdk4/6 inhibitor in combination with rapamycin-analog everolimus (ClinicalTrials.gov identifier NCT03070301). In another trial, a combination of the VEGFR2 inhibitor sunitinib and the c-met inhibitor PF-04217903 blocked tumor progression in RT2 mice^10^; subsequently, a PanNET patient clinical trial was initiated to test the effects of cabozantinib, a single agent targeting both VEGFR2 and c-met^11^.

The liver metastasis found in patients with PanNETs can also be detected in RT2 mice, although the frequency of metastasis is generally low. Researchers have published many reports on genes that can increase the rate of metastasis in this mouse, including *Igf1r*, *Alk*, *Rhamm*, *Met*, *Bclx*, and *C5* (refs. ^10,12–16^). Also, *Csf1* has been shown to be a metastasis suppressor in RT2 mice^17^.

Clinically, metastasis correlates with whether or not PanNETs produce insulin. PanNETs producing insulin are called insulinomas and these tumors are rarely malignant or metastatic; conversely, non-insulin-producing PanNETs are often highly malignant and metastatic^18^. Most of the non-insulin-producing PanNETs are “nonfunctioning” tumors (NF-PanNETs), so-named because they do not overproduce any of the major pancreatic endocrine hormones^18^. NF-PanNETs are by far the most clinically important of the pancreatic neuroendocrine tumors. It has been estimated that about 85% of PanNETs are nonfunctioning, 10% are insulinomas, and the remaining tumors express other hormones such as gastrin or glucagon^19^. Patients with nonfunctioning PanNETs have a 5-year survival rate of only 33%^19^, whereas patients with insulinomas rarely die of their disease. Nonfunctioning tumors are also larger in size than insulinomas.

Here we demonstrate that the RT2 mouse model is capable of modeling both insulinomas and nonfunctioning PanNETs, with the specific disease dependent on the genetic background of the animals. In a hybrid AB6F1 genetic background, low expression levels of a beta cell transcription factor, *Insm1*, favors development of nonfunctioning tumors, whereas in a C57Bl/6 genetic background, higher expression of *Insm1* favors development of insulinomas. Amounts of *Insm1* correlated with insulin production and with lack of metastasis, in both mice and patients.

## Results

**Fig. 1:**
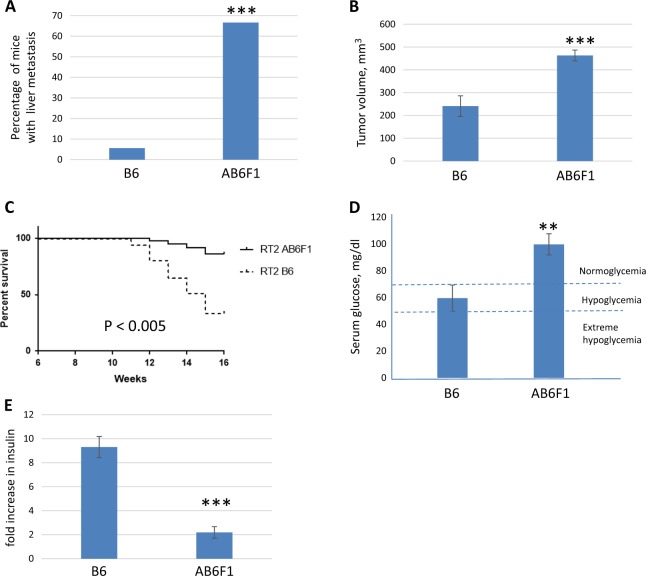
RT2 B6 and RT2 AB6F1 mice show profound phenotypic differences. **a** Percentage of 15-week-old RT2 mice with liver metastasis, by genetic background. All mice were males, and 18 mice from each lineage were analyzed. Statistical significance was determined using Fisher’s exact test. B6 mice have the C57Bl/6 genetic background, and AB6F1 mice are hybrids resulting from mating female A/J mice to male C57Bl/6 mice. **b** Volume of primary pancreatic tumors according to the genetic background. All mice were 17-week-old males; 24 RT2 B6 mice and 138 RT2 AB6F1 mice were analyzed. Statistical significance was determined using Mann–Whitney test. **c** Kaplan–Meier survival curves for RT2 mice between 6 and 16 weeks of age according to the genetic background. **d** Eight hours after removal of food, serum glucose was measured in RT2 mice of different genetic backgrounds. All mice were males and 12–13 weeks old. Fifteen RT2 AB6F1 and 12 RT2 B6 were analyzed. Statistical significance was determined using two-tailed *t*-test. **e** RT2 mice or wildtype littermates were held without food for 8 h, then serum insulin was measured and compared to littermates (B6 or AB6F1) lacking the SV40 T-antigen transgene. Mice were males and 13 weeks old. Nine and 11 RT2 B6 and RT2 AB6F1 mice were measured, respectively. Statistical significance was determined using two-tailed *t*-test

RT2 AB6F1 mice were generated by mating females from the inbred A/J genetic background to male RT2 B6 mice, which have an inbred C57Bl/6 J genetic background^12^. For both genetic backgrounds, pancreatic tumors were produced that were neuroendocrine tumors upon pathological examination, albeit less well-differentiated than the PanNETs found in most patients. There was a difference in frequency of liver metastasis in the two genetic backgrounds, with metastasis being much more common in RT2 AB6F1 mice than in RT2 B6 mice (Fig. 1a). Other genetic backgrounds have also been reported to influence metastasis frequency of RT2 mice^13^. Primary tumors were larger in age-matched RT2 AB6F1 mice compared to RT2 B6 (Fig. 1b). For RT2 AB6F1 animals, tumor size correlated with the presence of metastasis (Supplemental Fig. 1), while for RT2 B6 animals, a correlation between metastasis and tumor size was not tested because there were so few cases of animals with metastasis.

Remarkably, in spite of having larger, more metastatic tumors, RT2 AB6F1 mice actually lived longer than RT2 B6 mice (Fig. 1c). Eighty-five percentof RT2 AB6F1 mice but only 33% of RT2 B6 mice lived to an age of 16 weeks. Early mortality may be related to hypoglycemia, which could be observed only in RT2 B6 mice and not in RT2 AB6F1 mice (Fig. 1d). Hypoglycemia has previously been reported in RT2 mice, so it was actually a surprise to find out that the RT2 AB6F1 were not hypoglycemic. In keeping with their hypoglycemia, RT2 B6 mice were also severely hyperinsulinemic, with an average nine-fold increase in serum insulin compared to wildtype B6 mice (Fig. 1e). Conversely, in spite of their larger pancreatic neuroendocrine tumors, RT2 AB6F1 expressed only two-fold more serum insulin than wildtype AB6F1 mice. Immunohistochemistry revealed robust staining for insulin in tumors from RT2 B6 animals, but no insulin staining in tumors from RT2 AB6F1 animals (Supplemental Fig. 2).

**Fig. 2:**
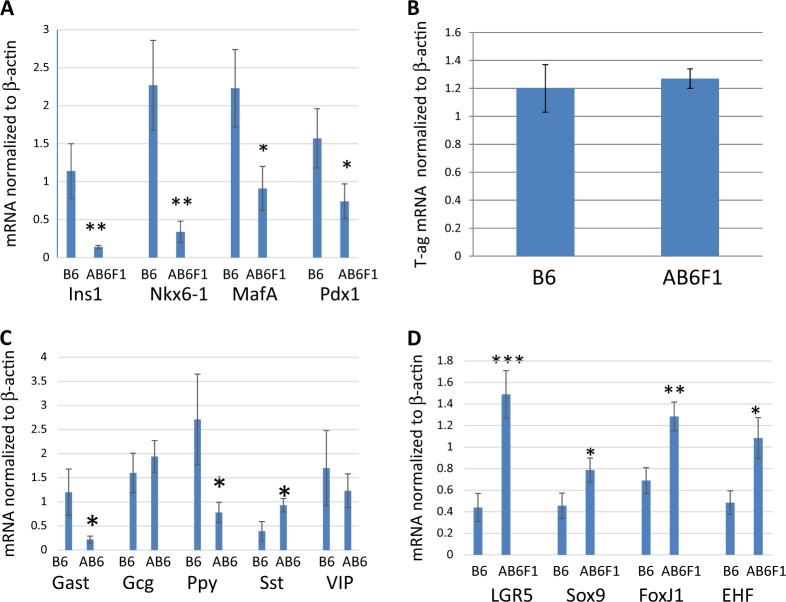
Tumors from RT2 AB6F1 mice profile as nonfunctioning pancreatic neuroendocrine tumors. In each figure, Q-PCR was used to measure gene expression, using cDNA prepared from primary tumors isolated from 17 male RT2 B6 mice and from 25 male RT2 AB6F1 mice. All statistical analysis was performed using two-tailed *t*-test. a Beta cell markers insulin-1 (*Ins1*), *Nkx6-1*, *MafA*, and *Pdx1*. **b** SV40 T-antigen, which is the oncogene that drives tumorigenesis in RT2 mice. **c** Pancreatic neuroendocrine hormones gastrin (Gast), glucagon (Gcg), pancreatic polypeptide (Ppy), somatostatin (Sst), and vasoactive intestinal peptide (Vip). **d** Pancreatic stem cell markers *Lgr5*, *Sox9*, *FoxJ1*, and *EHF*

Primary pancreatic tumors from both RT2 B6 and RT2 AB6F1 mice were harvested, and the latter expressed lower levels of mRNA for insulin (Fig. 2a), consistent with the low levels of serum insulin in these animals. Tumors from RT2 AB6F1 mice also transcribed less mRNA for other beta cell markers such as *MafA*, *Pdx1*, and *Nkx6-1* (Fig. 2a). Interestingly, transcription of the gene for SV40 T-antigen did not differ between tumors from RT2 AB6F1 and RT2 B6 mice (Fig. 2b), even though insulin expression differs strongly between the two mice. This may reflect the leakiness of the RIP that controls expression of SV40 T-antigen; this transgene has also been shown to express in other insulin-negative neuroendocrine cells from small intestine and pituitary^20,21^.

With their smaller tumors, low metastasis, hypoglycemia, and hyperinsulinemia, RT2 B6 mice have all of the clinical features of human insulinomas. RT2 AB6F1 mice, on the other hand, may develop some other kind of pancreatic neuroendocrine tumor that does not express insulin. In RT2 AB6F1 tumors, transcription of glucagon and vasoactive intestinal peptide were not elevated, transcription of PPY and gastrin decreased, but transcription of somatostatin increased (Fig. 2c). Although elevated, the levels of somatostatin transcription were still low. Patients with somatostatinomas suffer from weight loss and diarrhea, whereas RT2 AB6F1 were slightly overweight and had firm stools (data not shown). From these experiments, we conclude that tumors produced by RT2 AB6F1 mice profiled as nonfunctioning PanNETs, a clinically important disease in which patient tumors do not express high levels of any of the pancreatic hormones.

Thus in spite of sharing 50% genetic identity, RT2 AB6F1 and RT2 B6 mice develop two very different types of PanNET. A possible clue for the genetic basis of this difference came from analysis of transcription of several markers of pancreatic stem cells^22^, which increased in tumors from RT2 AB6F1 mice (Fig. 2d). The loss of differentiation (Fig. 2a) but gain in stem cell markers (Fig. 2d) in RT2 AB6F1 tumors led to a hypothesis that A/J and B6 mice may have different allelic forms of some beta cell differentiation factor.

Among the many genes known to be important for beta cell differentiation is *Insm1*, which can drive transdifferentiation of pancreatic ductal cells to endocrine cells^23^, presumably through a stem cell intermediate. *Insm1*-knockout mice have also been reported to make beta cells that are deficient in expression of insulin^24^, which is perhaps similar to the deficient expression of insulin in tumors of RT2 AB6F1 mice. Notably, *Insm1* was first isolated as a very highly expressed RNA in insulinomas^25^.

**Fig. 3:**
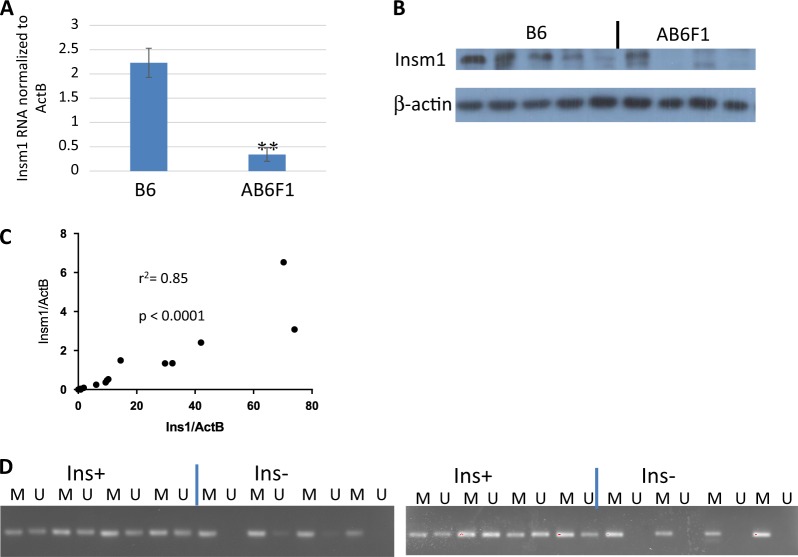
Insulinomas from humans and mice express high levels of transcription factor *Insm1*. **a** Q-PCR analysis of *Insm1* expression from primary tumors isolated from 17 male RT2 B6 mice or from 25 male RT2 AB6F1 mice. Statistical significance was determined using two-tailed *t*-test. **b** Western blot analyses of *Insm1* and β-actin expression from pancreatic neuroendocrine tumors of RT2 B6 or RT2 AB6F1 mice. **c** mRNA and cDNA were prepared from 39 human pancreatic neuroendocrine tumors, and transcription of beta cell markers *Ins1* and *Insm1* was assayed by Q-PCR. Most of the tumors occupy the (0,0) point of the graph. Statistical analysis was performed by Pearson correlation. **d** Tumor DNA from 16 patients was subjected to bisulfite treatment, followed by PCR using primers directed against a CpG island immediately upstream of the *Insm1* transcription start site. The results of PCR by *M*ethylation-specific primer pairs are shown in lanes marked “M”, and results of PCR by *U*nmethylation-specific primer pairs are shown in the lanes marked “U”. In both gels, the first four tumors had high insulin expression by Q-PCR analysis, and the second four tumors had undectable insulin expression by Q-PCR analysis. Tumors were arbitrarily defined as having high insulin expression if they had an *Ins1/ActB* mRNA ratio above 1.0 (see **c**). The eight insulin-negative tumors are likely to be nonfunctioning tumors, given the fact that 85% of PanNETs are nonfunctioning and 10% of PanNETs express insulin^19^. These eight insulin-negative tumors also had low expression of mRNA for gastrin and glucagon (data not shown)

As shown in Fig. 3a, expression of *Insm1* mRNA was higher in the insulinomas from RT2 B6 and lower in nonfunctioning tumors from RT2 AB6F1 mice. *Insm1* protein levels were also higher in tumors from RT2 B6, as shown by western blotting (Fig. 3b). These experiments suggested that *Insm1* might be relevant to the insulinomas and to the nonfunctioning tumors observed in RT2 mice.

*Insm1* expression was characterized within a large set of patient samples. As shown in Fig. 3c, the mRNA of *Insm1* strongly correlated with the mRNA of insulin. DNA was prepared from 16 of these human tumors, 8 of which expressed high amounts of insulin and 8 of which expressed very little insulin. Tumor DNAs were treated with bisulfite, and methylation-specific PCR was performed to test for DNA methylation within the promoter region of the *Insm1* gene. As shown in Fig. 3d, the *Insm1* promoter was more strongly methylated in the tumors with low insulin and low *Insm1*. Since promoter hypermethylation is a common mechanism for lowering expression of key tumor suppressor genes, these data suggest that *Insm1* expression may not merely correlate with insulinomas, but may actually be a gene that suppresses the formation of nonfunctioning PanNETs.

Next we tested whether allelic differences in the *Insm1* genes encoded by the B6 and A/J lineages might be responsible for the insulinomas and NF-PNETs observed in RT2 B6 and RT2 AB6F1 mice, respectively. *Insm1* genotypes were tested in a set of 279 RT2 AB6F2 mice, which had been prepared for a previous study^12^. RT2 AB6F2 are the product of mating RT2 AB6F1 mice. Unlike their hybrid F1 parents, which are genetically identical, F2 mice have a 1:2:1 Mendelian ratio of genotypes at all loci: one quarter of offspring will be homozygous for a given gene encoded by the A/J lineage, one quarter will be homozygous for the same gene encoded by the B6 lineage, and one half will be heterozygous at the locus. If a particular gene from the A/J genetic background promotes metastasis, then homozygotes and possibly heterozygotes for the A/J allele of that gene should be more likely to show liver metastasis. A known SNP difference between the *Insm1* alleles from C57Bl/6 and A/J mice was assayed.

**Fig. 4:**
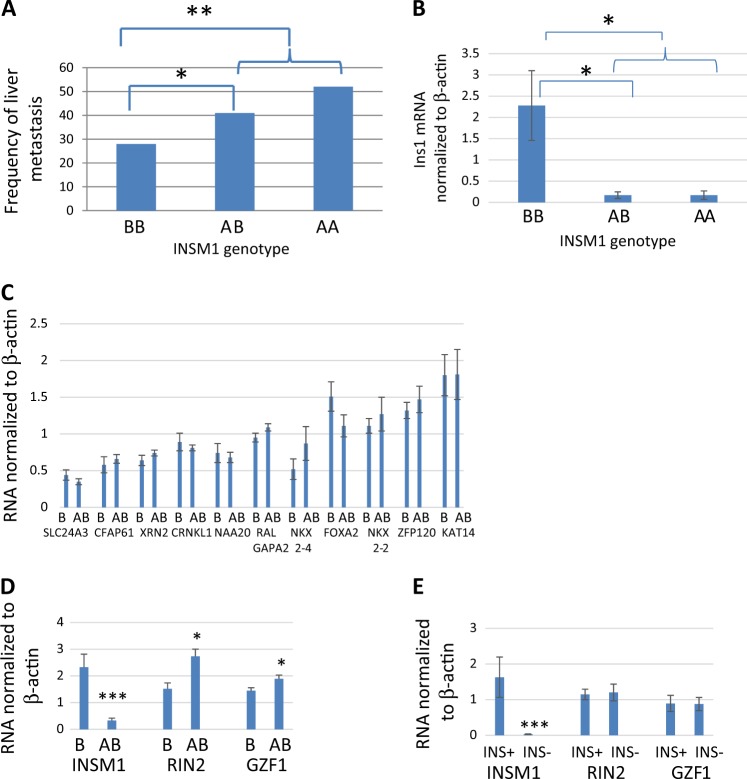
The *Insm1* locus links to key phenotypic differences between RT2 B6 and RT2 AB6F1 mice. **a** RT2 AB6F2 mice were genotyped for a nucleotide difference within the *Insm1* gene. Animals with two copies of the allele from the A/J lineage are called “AA”, animals with two copies of the allele from the B6 lineage are called “BB,” and heterozygous animals are called “AB”. The mice were also scored for the presence of liver metastasis. A total of 279 mice were assayed. Statistical significance was computed using Fisher’s exact test. **b** Transcription of insulin-1 (*Ins1*) by 37 primary PanNETs from RT2 AB6F2 mice was compared using Q-PCR. The tumors were also genotyped to determine the *Insm1* genotype. Tumors with two *Insm1* alleles from the B6 lineage are denoted “BB”, tumors from heterozygous mice are denoted “AB,” and tumors with two *Insm1* alleles from the A/J lineage are denoted as “AA”. Statistical significance was determined using two-tailed *t*-test. **c** Q-PCR was used to analyze transcription of genes linked to *Insm1* in primary PanNETs from 18 RT2 B6 males and from 24 RT2 AB6F1 males. The total size of the region was 8 MeB, with *Insm1* in the center. All genes within 2 MeB on either side of *Insm1* were assayed, as well as a select number of candidate genes within 4 MeB on either side of *Insm1*. Candidate genes were selected as genes that were known to encode transcription factors and differentiation factors. “B” refers to tumors from RT2 B6 mice and “AB” refers to tumors from RT2 AB6F1 mice. mRNA for several genes within this region, including *OvoL2*, *Kiz*, and *Pax1*, could not be detected in spite of using two separate assays for each gene. **d** Transcription of *Insm1*, *Rin2*, and *GZF1* in primary tumors from 18 RT2 B6 males and from 24 RT2 AB6F1 males was determined using Q-PCR. **e** Transcription of *Insm1*, *Rin2*, and *GZF1* was determined for 39 primary patient pancreatic NETs. Tumors were arbitrarily defined as expressing insulin or not expressing insulin based on Ins1/ActB mRNA ratios above or below 1.0, respectively (see Fig. 3c)

As shown in Fig. 4a, the *Insm1* SNP from the A/J background linked to metastasis in RT2 AB6F2 mice. Particularly notable is the fact that there was more metastasis in heterozygous mice compared to mice homozygous for the B6 allele of *Insm1*; this result matches with the fact that metastasis is more common in RT2 AB6F1 mice, which are heterozygous, than in RT2 B6 mice, which are homozygous for the B6 allele.

Importantly, if the differences in *Insm1* alleles are responsible for the different types of PanNETs, then the *Insm1* allele that links to metastasis should also link to lower insulin production. This is indeed the case. As shown in Fig. 4b, tumors from heterozygous animals expressed lower levels of mRNA for insulin than tumors from animals that inherited both of their *Insm1* alleles from the B6 lineage. Again, this matches with the lower levels of insulin produced by RT2 AB6F1 mice, which are heterozygous, and with the higher levels of insulin produced by RT2 B6 mice, which are homozygous for the B6 allele. Thus, in F2 animals with at least one copy of *Insm1* from the A/J lineage, there was elevated metastasis and weak expression of insulin when compared to animals with two copies of *Insm1* from the B6 lineage.

We also considered the possibility that a gene linked to *Insm1*, and not *Insm1* itself, is the actual effector of tumor type in RT2 mice. If true, then perhaps such a gene would show expression differences in RT2 B6 and RT2 AB6F1 tumors. Among genes residing within an 8 MeB region of the mouse genome, centered at *Insm1*, three genes did show differences in expression between tumors from RT2 B6 and RT2 AB6F1 mice (Fig. 4c, d). However, of these three genes, only *Insm1* expression also showed differences in patient samples expressing high or low levels of insulin (Fig. 4e). Although this study does not completely rule out the possibility that another gene is involved, *Insm1* does appear to be the best candidate.

**Fig. 5:**
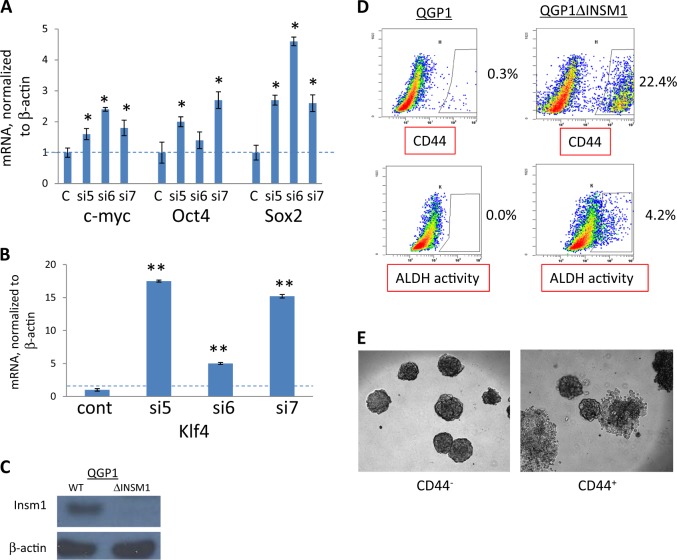
Loss of *Insm1* expression promotes an invasive stem cell phenotype in human pancreatic neuroendocrine tumor cell line QGP1. **a**, **b** The QGP1 cell line was transfected with three different siRNAs directed against *Insm1* (si-5, si-6, or si-7) or with control si-RNA (C, or cont). Eighty-four hours later, transcription of reprogramming genes *Myc*, *Oct4*, and *Sox2* (**a**) or *Klf4* (**b**) was measured by Q-PCR. **c** Western blot results, using an antibody against *Insm1* to measure expression in the QGP1-TGL cell line, and in an isogenic version of QGP1-TGL in which the *Insm1* genes were knocked out using CRISPR-Cas9. Sequencing results for the knockout cell line are shown in the Materials and methods section. **d** The QGP1-TGL cell line expressing *Insm1*, as well as QGP1-TGL stably knocked out for the *Insm1* gene, were tested for expression of stem cell markers CD44 and aldehyde dehydrogenase using flow cytometry. **e** Fluorescence activated cell sorting was used to collect CD44-positive and CD44-negative QGP-TGL cells, which were also knocked out for *Insm1*. The sorted cells were used to grow three-dimensional colonies in matrigel. CD44-negative cells formed sharply demarcated spheroids, but the colonies formed by CD44-expressing cells often lost their spheroid shape and appeared to invade the matrigel

If alleles of *Insm1* are indeed responsible for the two tumor subtypes, then *Insm1* should be capable of blocking metastasis and dedifferentiation in neuroendocrine tumor cell lines. In Fig. 5a, b, the effect of transient knockdown of *Insm1* expression using three different shRNAs was tested in the QGP1 cell line. *Insm1* knockdown increased expression of the so-called Yamanaka factors^26^, which are known to increase pluripotency of differentiated cells. Next, CRISPR-Cas9 technology was used to stably knock out *Insm1* from QGP1-TGL cells, a version of QGP1 engineered to express firefly luciferase (Fig. 5c). The *Insm1* knockout increased the number of cells expressing aldehyde dehydrogenase and CD44, which are markers of stem cells (Fig. 5d). *Insm1*-null QGP1-TGL cells were then sorted for CD44 expression using flow cytometry. CD44-negative QGP1-TGLΔInsm1 cells gave rise to spheroid colonies, whereas CD44-positive QGP1-TGLΔInsm1 cells produced colonies with invasive morphologies (Fig. 5e). These data indicate that loss of *Insm1* can produce more invasive, stem-like cells.

**Fig. 6:**
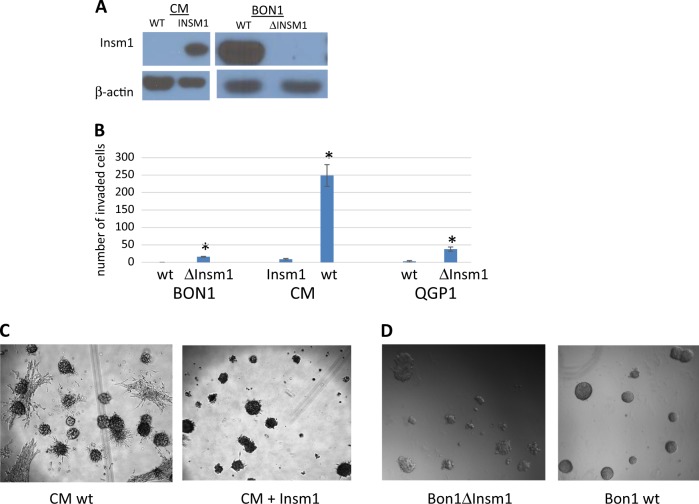
*Insm1* expression decreases invasiveness of human pancreatic neuroendocrine tumor cell lines. **a** Western blot analysis, using antisera against *Insm1* or β-actin to analyze protein extracts from isogenic pairs of two cell lines (CM and BON1) in which *Insm1* was either expressed or not expressed. BON1 normally expresses *Insm1*, so the two alleles were removed using CRISPR. CM cells do not normally express *Insm1*, so a plasmid expressing *Insm1* under control of the CMV promoter was introduced and a clone with physiological expression of *Insm1* was isolated. **b** Transwell assays were used to measure the ability of cells to migrate toward 20% serum across a membrane with 8 μm pores, and then to invade matrigel. The tested cell lines were wildtype (wt) BON1, QGP1-TGL, or CM, along with isogenic versions of the cell lines that were engineered to alter their ability to express *Insm1*. **c** Single cells of the highly invasive CM cell line were grown into three-dimensional colonies for 2 weeks in matrigel. The presence of *Insm1* changes the morphology of the colonies, decreasing their invasiveness and turning them spheroid. **d** Single cells of the BON1 cell line were grown into three-dimensional colonies for 2 weeks in matrigel. The cells deleted for *Insm1* have a less spheroid, more invasive morphology compared to wildtype cells, although this difference was not as dramatic as observed for CM in **c**

CRISPR-Cas9 was also used to knock out expression of *Insm1* from a second *Insm1*-expressing PanNET cell line, BON1 (Fig. 6a). We also introduced *Insm1* expression to the CM cell line, a highly undifferentiated PanNET cell line that did not normally express *Insm1* protein (Fig. 6a). Along with QGP1-TGL, each of these isogenic pairs of *Insm1*-expressing and *Insm1*-null cell lines was tested for in vitro invasiveness, using a transwell assay. Invasiveness is a common property of metastatic cells. For all of the cell lines, invasiveness was higher if expression of *Insm1* was absent, indicating that *Insm1* is a repressor of invasion (Fig. 6b). The CM cell line was particularly invasive; the effects of *Insm1* on this cell line could also be seen in three-dimensional culture, in which wildtype CM cells invade the surrounding matrix, but CM cells expressing *Insm1* only form less invasive spheroids (Fig. 6c). Though less dramatic, *Insm1* also has an effect on the morphology of the BON1 cell line when grown in three-dimensional culture (Fig. 6d); the smaller change in morphology of BON1 correlated with the lower level of invasiveness of BON1 cells compared to CM cells.

**Fig. 7:**
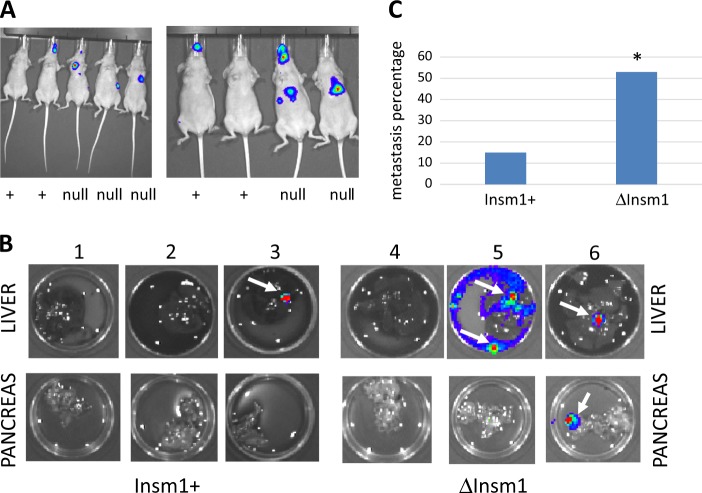
*Insm1* blocks in vivo metastasis by the QGP1-TGL cell line. **a** The QGP1 cell line was engineered to express firefly luciferase, and isogenic pairs either expressing *Insm1*, or knocked out for *Insm1*, were injected into the left ventricles of nude mice. Three weeks later, metastatic lesions were imaged in live mice following injection of luciferin. In both photos, the two mice on the left were injected with cells that express *Insm1* (+), and the other animals were injected with cells unable to express *Insm1* (null). **b** Three weeks after intracardial injection of luciferase-expressing QGP1 cells, mice were euthanized, and pancreas and liver were removed and treated with luciferin to help image metastases. The first three mice were injected with *Insm1*-expressing QGP1, and mice 4, 5, and 6 were injected with *Insm1*-negative QGP1. Livers are in the upper row and pancreases are in the lower row. Metastases are noted by arrow. **c** Quantitation of metastases from lung, pancreas, and liver, which were isolated from 14 mice treated with luciferase-expressing QGP-TGL cells that either express or cannot express *Insm1*. Metastasis was arbitrarily defined as individual organs giving radiance readings above 10^5^ photons/s/cm^2^/steradian following luciferin treatment. Statistical significance was determined using Fisher’s exact test

Isogenic, *Insm1*-expressing, or *Insm1*-null QGP1-TGL cells were injected into the left ventricles of immune compromised mice. Mice were imaged weekly for bioluminescence of luciferase in order to assay for metastatic spread. Within 2 weeks, strong luciferase signals were detected in the torso, and these were particularly intense in animals injected with *Insm1*-null QGP1 (Fig. 7a). Luciferase signals also occurred above the neck; magnetic resonance imaging of the head revealed unusual growth of cells in the pre-nasal sinuses. Dissection of animals revealed that metastatic lesions were most prominent in the lung, liver, and pancreas. Examples are shown in Fig. 7b, where one of three animals injected with wildtype QGP-TGL produced a small lesion in the liver (animal number 3), but two of three animals (animals 5 and 6) injected with QGP-TGL knocked out for *Insm1* produced lesions in the pancreas and in the liver. Altogether, the presence of metastatic tumors was 3.3-fold more common in the animals injected with the cell line knocked out for *Insm1* (Fig. 7c).

Together, these cell line experiments establish that depletion of *Insm1* can cause stem-like cells to form, can increase invasiveness, and can cause metastasis; furthermore, these data suggest that the difference between the less metastatic, more differentiated insulinomas of RT2 B6 mice and the NF-PanNETs of RT2 AB6F1 mice may be due to differences in *Insm1* expression. Coupled with the genetic linkage experiments from RT2 AB6F2 mice, the cell line data strongly suggest that allelic differences in *Insm1* between the A/J and B6 mouse lines lead to different types of neuroendocrine tumors in RT2 mice.

## Discussion

In two different genetic backgrounds (B6 and AB6F1), RT2 mice produced different types of PanNETs: insulinomas and nonfunctioning PanNETs, respectively. Several lines of evidence supported the idea that allelic differences in the *Insm1* gene were responsible for the difference in tumor types, and that *Insm1* is a suppressor gene of nonfunctioning PanNETs. First and most importantly, in RT2 AB6F2 mice, the *Insm1* locus linked strongly to high metastasis and to low insulin expression, which are two key characteristics of NF-PanNETs. Second, *Insm1* expression was lower in NF-PanNETs, compared to insulinomas. Third, the *Insm1* promoter was hypermethylated in human NF-PanNETs, as is often observed for tumor suppressor genes. Fourth, a key feature of NF-PanNETs is metastasis, and knockout of *Insm1* was sufficient to increase in vivo metastasis of a pancreatic neuroendocrine cell line, QGP1-TGL1, and to increase in vitro invasiveness of additional neuroendocrine cell lines.

*Insm1* is a transcription factor that promotes beta cell differentiation^27^, and other tissue-specific differentiation factors have also been shown to suppress metastasis, such as GATA3 in breast tumors^28^ and NKX2-1 in lung tumors^29^. It is generally thought that loss of these differentiation factors leads to metastasis by causing dedifferentiation to stem cells, and that stem cells increase metastasis because they are more invasive and less prone to anoikis^30–32^. Expression of stem cell markers increased in low *Insm1*-expressing tumors from RT2 AB6F1 mice (Fig. 2d). Upon knockout of *Insm1* in the QGP1 cell line, stem cell markers increased, invasiveness increased, and metastasis increased.

The A/J and B6 lineages show several nucleotide differences within and near the *Insm1* locus, and it is possible that one or more of these differences affect *Insm1* transcription or RNA stability. It is also formally possible that the *Insm1* alleles in these lineages are not different at all and that another gene, linked to *Insm1*, can affect *Insm1* expression. But we detected no evidence for such a mechanism, such as differential expression of another linked gene.

Interestingly, *Insm1* is not the only allelic difference between the B6 and A/J lineages that affects neuroendocrine tumors. In our initial study of RT2 AB6F2 mice, we showed that the A/J genome harbored a recessive, metastasis-suppressing locus, due to an inactivating mutation in the gene for *C5* (ref. ^12^). But given that mating RT2 B6 with A/J mice produced an F1 generation with a very large increase in metastasis (Fig. 1a), we thought that the A/J genome might also contain a metastasis-promoting locus that was either dominant or co-dominant, and this allele turned out to be *Insm1*. Additionally, it has been shown that the *Alk* allele encoded by the B6 genome promotes metastasis more strongly than the *Alk* allele encoded by the C3H inbred line^13^, and we also have evidence that the B6 allele of *Alk* promotes metastasis more strongly than the *Alk* allele of the A/J background (Contractor and Harris, unpublished data). Lastly, we have genetic data that metastasis in RT2 mice is also affected by yet another allelic difference between the A/J and B6 lineages, but have not yet identified the actual gene (Contractor and Harris, unpublished data). Thus four, and possibly more, naturally occurring differences between the A/J and B6 mouse lines have a strong effect on whether the animal has a localized tumor or a metastatic one, an insulinoma or a NF-PanNET. Small-nucleotide polymorphisms that affect tumorigenesis have been identified for many tumor types^33,34^, but the high number of natural differences between A/J and B6 that affect neuroendocrine tumorigenesis in RT2 mice makes one wonder whether NETs are peculiarly susceptible to naturally occurring genetic diversity.

The subtypes of pancreatic neuroendocrine tumors that can occur in patients show important clinical differences, with nonfunctioning PanNETs being more common and causing worse outcomes than insulinomas^19^. Understanding how different tumor subtypes can occur has been an active research area. In animal models for breast and brain tumors, it has been demonstrated that certain oncogene or tumor suppressor mutations can favor development toward one tumor subtype and not another^35,36^. In recently published work, mutations in *Men1*, *Daxx*, and *Atrx* were shown to be less common in human insulinomas than in human nonfunctioning tumors, suggesting that subtypes of PanNETs are influenced by the presence of these driver mutations^37^. But the present work also shows that tumor suppressor gene mutations are not sufficient to explain why different tumor subtypes can occur. Using mice that share a common driver oncogene, we show that tumor subtype is also influenced by naturally occurring genetic diversity, in the form of distinct alleles of the *Insm1* gene. It would be interesting to know whether *Insm1* expression can be directly influenced by mutations in *Men1*, *Daxx*, and *Atrx*, which encode chromatin-remodeling proteins. However, it should also be stated that mouse models do not always reflect how tumors develop in patients; indeed it is not clear whether low *Insm1* expression is a direct cause of nonfunctioning PanNETs in patients, or a byproduct of the cell type from which these tumors originate^38^.

In patients, a key feature of NF-PanNETs is high rate of metastasis; NF-PanNETs in RT2 AB6F1 mice are likewise highly metastatic. The development of this mouse model may improve the ability to test drugs that could prevent metastasis of PanNETs in vivo. The RT2 mouse has previously been used for in metastasis studies, but comes with important shortcomings: metastasis is less penetrant in RT2 B6 mice, and is complicated by early death due to hypoglycemia. The RT2 AB6F1 mouse overcomes these issues. Metastasis is rapid and highly penetrant especially in male RT2 AB6F1 mice^12^, and RT2 AB6F1 mice are not hypoglycemic (Fig. 1d).

Several drugs have shown anti-metastatic activity in mouse models, but there have been no clinical trials designed to test drugs that might slow the risk of metastasis. This is in spite of the fact that metastasis is thought to be the cause of nearly all cancer deaths. There are several pragmatic reasons for the lack of trials on metastasis, including the fact that most clinical trials are performed on late stage, post-metastatic patients in order to overcome the high expense of lengthier trials on earlier stage patients. But another problem may be that spontaneous, synchronized mouse models of metastasis are rare. For this reason, the RT2 AB6F1 mouse model may be of great value. It derives from a mouse in which preclinical successes have led to human clinical trials and to FDA approvals. Although there is not a large population of patients with PanNETs, clinicians who study this disease are highly organized and have shown the ability to perform clinical trials that result in FDA approvals in spite of small patient sets. Patients with PanNETs are often detected before their tumors are metastatic, yet the onset of metastasis is fairly rapid for patients with nonfunctioning disease. All of these factors—a rapid and validated mouse model, a set of early diagnosed patients, fairly rapid onset of metastasis, and an organized set of clinicians—suggest that the RT2 AB6F1 mouse could prove to be an attractive preclinical model for developing and testing clinically relevant anti-metastatic agents.

## Materials and methods

### Cell lines

The QGP1 cell line was purchased from the Japan Health Sciences Foundation. The BON1 cell line was a gift from the lab of Kjell Oberg and the CM cell line was a gift from the lab of Paolo Pozzilli. In order to authenticate the cell lines, short tandem repeat analysis was performed by ATCC. The STR results for QGP1 were a 100% match to STR information for QGP1 found in the DSMZ database. The STR results for BON1 were a 100% match to previously published information^39^. STR results for CM did not match any other cell lines in the databases for ATCC, DSMZ, or EXPASY. STR results for the CM cell line have not previously been published, and are presented in Supplemental Fig. 3. QGP1 and BON1 have previously been tested for mycoplasma but CM cells were not. CM and QGP1 were grown in RPMI media (Thermofisher) supplemented with 10% fetal bovine serum (Sigma-Aldrich). BON1 cells were grown in Dulbecco’s modified Eagle’s medium (Thermofisher) with 10% fetal bovine serum. All lines were grown at 37 °C under 5% CO_2_. To decrease heterogeneity, the cell lines were cloned from single colonies before use. CM cells were engineered to express human *Insm1* by using lipofectamine 2000 (Thermofisher) to transfect a plasmid expressing the human gene under control of the CMV promoter (Origene); *Insm1*-expressing cells were cloned from single transfectants. QGP1 and BON1 were engineered to express luciferase, and then *Insm1* was knocked out by CRISPR, using an EDIT-R lentivirus purchased from Dharmacon (source clone identification number VSGHSM_26789438). Potential low expressers were cloned, and characterized by RTPCR and by western blotting, using an *Insm1* antibody that was a generous gift from Mark Magnuson. Antisera against β-actin (part number A2228) were purchased from Sigma. Genomic DNA from low *Insm1*-expressing clones was subjected to PCR using primers 5′-CAGGTGTTCCCCTGCAAGTA and 5′-CCCAGACAACAGTTCAAGGC; the PCR products were cloned using the TOPO TA system (Thermofisher), and 12 of the PCR clones were sequenced to confirm the presence of frameshift mutations within the *Insm1* sequence targeted by the EDIT-R lentivirus. BON1 clone 145 showed two new alleles of *Insm1*, each of which had frameshifting deletions: loss of the 10 nt sequence **ctcgcccggca**, or loss of a single nucleotide, shown emboldened and in lower case within the following sequence: CCCGGC**c**TTACG. QGP1 clone H10 also showed two new alleles of *Insm1*, each of which had frameshifting mutations: loss of two nucleotides, CCCGGC**ct**TACGCG, or loss of a single nucleotide, CCCGGC**c**TTACGCG. A matrigel-coated invasion chamber with 8 μm pore size (Corning 354480) was used for invasion assays; 20% fetal bovine serum in the upper chamber was used as a chemo-attractant. Three-dimensional growth of cell lines was performed by mixing 1000 cells with 50 μl of matrigel (Corning 354234), and seeding 5 μl of this mixture into 96-well plates. After 5 min, 200 μl of RPMI/10% FBS media were added and the cells were cultured under CO_2_ at 37 °C for 2 weeks. *Insm1* siRNAs si-5, si-6, and si-7 were purchased from Dharmacon, and catalog numbers were J-006535-05-002, J-006535-06-002, and J-006535-07-002, respectively

### Human and mouse experiments

Human pancreatic neuroendocrine tumors were provided by the Cooperative Human Tissue Network (CHTN), which obtained informed consents from patients. Human experiments were approved by the Institutional Review Board of CHTN. Mouse experiments were approved by the Institutional Animal Care and Use Committee of Rutgers University. RT2 B6 mice were obtained from the National Cancer Institute (Frederick, MD) and bred to C57Bl6/J (Jackson laboratories) for more than 10 generations. A/J mice were purchased from Jackson Laboratories. Mouse husbandry, euthanasia, and autopsy protocols have been previously described^12^. *Insm1* genotypes were not assayed until long after the mice had been euthanized and the metastasis and tumor size data were collected. To assay metastasis of the QGP1 cell line, one million cells expressing luciferase were injected into the left ventricles of nude mice, as previously described^14^. The day after surgery, and weekly thereafter, mice were sedated with isofluorane, injected with luciferin, and imaged using an IVIS machine. Three weeks after injection, the mice were euthanized by CO_2_ asphyxiation and cervical dislocation, and autopsied for metastatic lesions. Organs were examined visually for metastatic lesions. Three organs (liver, pancreas, and both lungs) were also removed, treated with luciferin, and imaged using an IVIS machine. To assay serum insulin and serum glucose, mice were moved to fresh cages with water bottles but no food for 8 h. An ELISA kit for mouse insulin was purchased from RayBiotech and used as recommended by the manufacturer. Serum glucose was measured using a ReliOn Confirm glucose meter and ReliOn Micro Plus test strips

### Flow cytometry analysis and cell sorting

Cells were incubated with PE-labeled CD44 antibody (Biolegend) and 7-AAD Viability Dye (Beckman Coulter). Activity of aldehyde dehydrogenase was determined with AldeFluor Kit (Stem cell Technologies). Flow cytometry analysis and cell sorting were performed using a Cytomics FC500 Flow Cytometer (Beckman Coulter) or a BD Influx High Speed Cell Sorter (BD Biosciences). The purity of sorted cells was >95%.

### Genotyping

An SNP assay directed against rs33272877, which differs between the *Insm1* alleles of A/J and C57Bl/6 genomes, was designed and synthesized by Thermofisher. Outside primer sequences were 5′-CGTGCTGGGCCTGAGT and 5′-GGGCGCCCTTGCT. Reporter sequences were 5′-ACCGCCCAGTGCCA and 5′-CCGCCGAGTGCCA. SNP analysis was perfomed using a Prism 7500 (Applied Biosystems).

### Analysis of RNA and protein

Fresh-frozen tumor tissues from male mice were minced in Trizol (Thermofisher) and homogenized using a Polytron 1200E. Chloroform was then added and the upper aqueous phase was removed after microcentrifugation at 14,000 r.p.m. Ethanol was added and the RNA was then isolated using an RNAeasy column (Qiagen) as recommended by the manufacturer. RNA was converted to cDNA using reverse transcription reagents (Thermofisher). Real-time RTPCR was then performed, using a Prism 7500 (Applied Biosystems). Most of the RNAs were measured using pre-designed Taqman assays, which were purchased from Thermofisher. The following assays were designed with Primer3 (http://bioinfo.ut.ee/primer3/) and run with SYBR green reagents (Thermofisher): mouse *Slc24a3* (5′-CCCTCTGGCAAACTGGAAAC and 5′-GGGATCCCTAGTGTGTAGCC); mouse *Cfap61* (5′-AATCACTACCCTCAGCTGCA and 5′-GCCAAAGAAGCATGACCCAT); mouse *Rin2* (5′-AACTCCTGGACCCATCATG and 5′-CATCCGCTGTTGACCTCTTG); mouse *Xrn2* (5′-GAGGTCAAGCTCAGATCCCAAA and 5′-GTTCCATGGCAGTAGAGGTTCA); mouse *Crnkl1* (5′ AGAGAAGAAAGGTCCAGGCC and 5′-GCTTGAGGTTAGGCTGGTTG); mouse *Naa20* (5′-AGGGAAGAATGGCATGGACA and 5′-GCTTGTACATGTTGACGGCA); mouse *Nkx2-4* (5′-GCCCCATGAACCTGGAGATT and 5′-CACCTACCACATGCCTCCC); human *Rin2* (5′-TCCGCACCATCTCCTGTTTC and 5′-GTCTGGACAAGCGAGGAAGT); mouse *Sox9* (5′-TATCTTCAAGGCGCTGCAAG and 5′-CCCCTCTCGCTTCAGATCAA); mouse *EHF* (5′-GCCCGGCAGAAAGTCTTACT and 5′-TTCCAGTCCGCACACAATGT); mouse *FoxJ1* (5′-CACTCTCATCTGCATGGCCA and 5′-AGGTTGTGGCGGATGGAATT); mouse *Kat14* (5′-ACGAGAGGCTGAAACTGACA and 5′-ACGTCCACTTCCTTCCAGAG); mouse *Zfp120* (5′-AAGCCCAGAAGTTCCGACAT and 5′-AGCAGCGAGATTCCTGTAGG); mouse *GZF1* (5′-CTCAGCGCAATTCCCTGTAC and 5′-GTGAACTGCTTCCCACACTG); and human *GZF1* (5′-TCACTCAGAACCACATGCTG and 5′-AATTCCGCTGGGCAAAAGTC.

### Analysis of promoter methylation

Tumor DNA was treated using EpiMark Bisulfite Conversion Kit as recommended by the manufacturer (New England Biolabs). Primers were designed using MethPrimer 1.0 (ref. ^40^). Methylation-specific primers were 5′-TTTATTTTACGCGGTTTATTTTTC and 5′-ATCGAATCGAAATATTTATCTTCG. Unmethylation-specific primers were 5′-TGTTTGTTTTTATTTAATTAGTG and 5′-TCAAATCAAAATATTTATCTTCACC.

### Immunohistochemistry

For antigen retrieval, slides were boiled for 16 min in 0.93% (v/v) Antigen Unmasking Solution H3301 (Vector Labs), and then slowly cooled to room temperature for 30 min. Primary antibody was guinea pig anti-insulin from Dako (A0564), which was diluted 2000-fold in 10% goat serum/1% BSA. Slides were treated with primary antibody overnight at 4 °C. Secondary antibody was affinity-purified, biotinylated anti-guinea pig IgG from Vector Labs (BA-7000), which was diluted 500-fold in 10% goat serum and 1% BSA. Slides were then treated with Vectastain Elite ABC peroxidase kit (Vector Labs) for 30 min, and with ImmPact DAB peroxidase substrate (Vector Labs SK-4105) for 1min. The slides were counterstained with hematoxylin (Vector Labs).

### Statistical analysis

Graphpad Prism 7.04 software was used for statistical analysis. Two-tailed *t*-test was used to compare RNA expression levels from sets of mouse and human tumors. Fisher’s exact test was used to compare metastasis frequencies. Pearson correlation analysis was used to compare *Insm1* and Ins1 expression in human tumors. Nonparametric Mann–Whitney analysis was used to evaluate tumor volumes. Outliers were identified by Rout analysis (*q* = 1%).

## Supplementary information

Supplemental figure legends

Supplemental Figures
