# Supplementary material for: Alleles of Insm1 determine whether RIP1-Tag2 mice produce insulinomas or nonfunctioning pancreatic neuroendocrine tumors
Source: Oncogenesis. 2019 Feb 22;8(3):16. doi: 10.1038/s41389-019-0127-1 (PMC6386750; doi:10.1038/s41389-019-0127-1)
Supplement: Supplementary file 1 — Supplemental figure legends [file 41389_2019_127_MOESM1_ESM.docx]

**Supplemental Figure Legends**

Supplemental Figure 1. **Primary tumor volumes correlate with presence of liver metastasis**. The sizes of primary tumors from 17-week-old RT2 AB6F1 animals were determined, and binned into two groups. The 53 animals in the first group showed no liver metastasis, while the 71 animals in the second group did show liver metastasis. Each data point represents a single animal. Statistical significance was determined by Mann Whitney test.

Supplemental Figure 2. **Tumors from RT2 B6 mice express higher amounts of insulin than tumors from RT2 AB6F1 mice.** Immunohistochemical analysis of insulin expression is shown for representative tumors from RT2 B6 and from RT2 AB6F1 mice. Each tumor is a primary pancreatic lesion, which was formalin-fixed and paraffin-embedded, then processed for immunohistochemistry by treating with an antibody generated against insulin. Brown staining indicates insulin expression, which is strong in the RT2 B6 tumors but absent in the RT2 AB6F1 tumors.

Supplemental Figure 3. **Short tandem repeat analysis of the CM cell line.** Human pancreatic neuroendocrine tumor cell line CM was subjected to STR analysis. The CM cell line is not in any of the publically available databases, and has not been previously subjected to STR analysis. The STR results presented here did not match any of the cell lines listed in the ATCC or DSMZ databases.
