# Supplementary material for: Alleles of Insm1 determine whether RIP1-Tag2 mice produce insulinomas or nonfunctioning pancreatic neuroendocrine tumors
Source: Oncogenesis. 2019 Feb 22;8(3):16. doi: 10.1038/s41389-019-0127-1 (PMC6386750; doi:10.1038/s41389-019-0127-1)
Supplement: Supplementary file 2 — Supplemental Figures [file 41389_2019_127_MOESM2_ESM.pptx]

## Slide 1
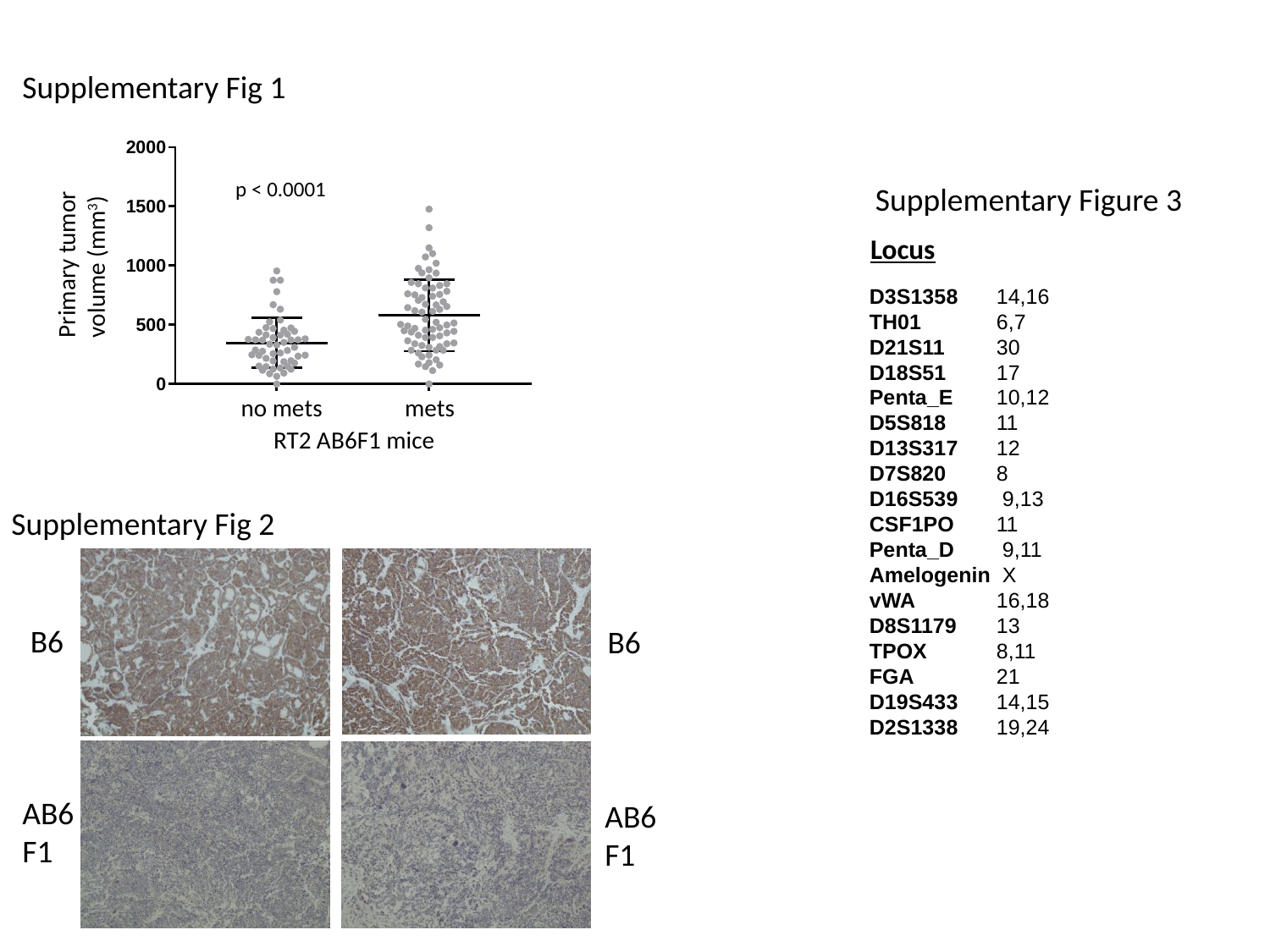

Supplementary Fig 1
p < 0.0001
Supplementary Figure 3
Primary tumor volume (mm3)
Locus
D3S1358 	14,16
TH01 	6,7
D21S11 	30
D18S51	17
Penta_E 	10,12
D5S818 	11
D13S317 	12
D7S820 	8
D16S539	 9,13
CSF1PO 	11
Penta_D	 9,11
Amelogenin	 X
vWA 	16,18
D8S1179	13
TPOX 	8,11
FGA 	21
D19S433	14,15
D2S1338	19,24
no mets mets
RT2 AB6F1 mice
Supplementary Fig 2
B6
B6
AB6F1
AB6F1
